# Development of Quantum dot-based enzyme biosensor for the detection of dopamine in urine

**DOI:** 10.1038/s41598-026-42466-3

**Published:** 2026-03-12

**Authors:** Dhaarini Sakharayapatna Yogaraju, Nidhi S. Shetty, Suhana Mohideen, Shama Prakash K, Akshath Uchangi Satyaprasad

**Affiliations:** 1 Department of Bio and Nanotechnology, Nitte University Centre for Science Education and Research-NUCSER, Nitte (Deemed to be University), Mangalore, Karnataka India; 2 Department of Biochemistry, KS Hegde Medical Academy (KSHEMA), Nitte (Deemed to be University), Paneer Campus, Kotekar-Beeri Road, Deralakatte, Mangalore, 575018 Karnataka India; 3 Department of General Medicine, KS Hegde Medical Academy, Nitte (Deemed to be University), Mangalore, Karnataka India

**Keywords:** Dopamine detection, Quantum dots, Enzyme biosensors, Fluorescence quenching, Biochemistry, Biological techniques, Biomarkers, Neuroscience

## Abstract

Dopamine (DA), a neurotransmitter released by the hypothalamus, plays a significant role in maintaining mental well-being. Abnormal DA level leads to neurological disorders such as depression and schizophrenia. Consequently, DA is commonly monitored in urine using various analytical methods as a non‑invasive approach for assessing its physiological status. The available methods for DA detection are laborious and time-consuming. To circumvent this issue, we developed a Quantum dot-based enzyme biosensor for the rapid, sensitive detection of DA. Fluorescence quenching of QDs was in proportion with the DA concentration and was found to be linear with an R^2^ = 0.99, with *p* < 0.05. The biosensor used to detect DA in urine samples in the range of 1.2 µM-8 µM, with R² = 0.97 and *p* < 0.05, and a limit of detection (LOD) of 1.2 µM in a urine sample (1:100). Spiking and recovery analysis in urine showed 94–98% recovery with *p* < 0.05. The developed method showed specificity towards detecting DA in the presence of common interfering factors such as uric acid and ascorbic acid. The results show that dopamine-specific quenching is consistent and concentration-dependent, effectively distinguishing the target from background components in complex samples. This approach provides a promising platform for reliable DA monitoring in clinical diagnostics.

## Introduction

Dopamine (DA), [4-(2-aminoethyl) benzene-1,2-diol], a neurotransmitter and a “feel-good hormone,” is synthesized primarily in the brain in the ventral tegmental area, substantia nigra, and hypothalamus^1^. It plays a significant role in the central nervous system, and abnormal levels of DA can lead to some vital neurological diseases like schizophrenia, Parkinson’s disease, and depression^2^. DA synthesis occurs via a two-step enzymatic reaction in which L-Tyrosine is converted to L-DOPA by tyrosine hydroxylase (TH). A further decarboxylation reaction catalyzed by DOPA decarboxylases produces DA^1^. The enzyme TH regulates DA biosynthesis, as well as that of other catecholamines. Hence, it is a promising approach for gene therapy and related treatment modalities^1^. Considering an average of 2 L of urine produced per day, it corresponds to 0.2–0.4 µmol/L^3^. In addition to these biological fluids, DA levels were monitored non-invasively in tear fluid. Schirmer’s strips and capillary tubes are utilized to extract tears and measure DA levels^4^. Recent studies and techniques used to detect DA include conventional liquid chromatography^5^, electrochemical detection coupled with high-performance liquid chromatography (HPLC)^6^, microdialysis with capillary liquid chromatography^7^, colorimetric^8,9^, enzyme-based methods^10^, enzyme-linked immunosorbent assay (ELISA)^11^, etc. Due to the presence of DA at lower concentrations in body fluids, a highly sensitive detection method is often required. Long chromatographic run times, lack of specificity in the colorimetric method for detecting structurally similar catecholamines, cross-reactivity, enzyme instability, the need for skilled personnel, and the requirement for advanced instrumentation make the currently available techniques less reliable and efficient. Hence, biosensors developed using fluorescent tags or nanomaterials, which address the limitations of conventional methods, can improve the robustness, specificity, and sensitivity of detection^12–14^.

**Scheme 1:**
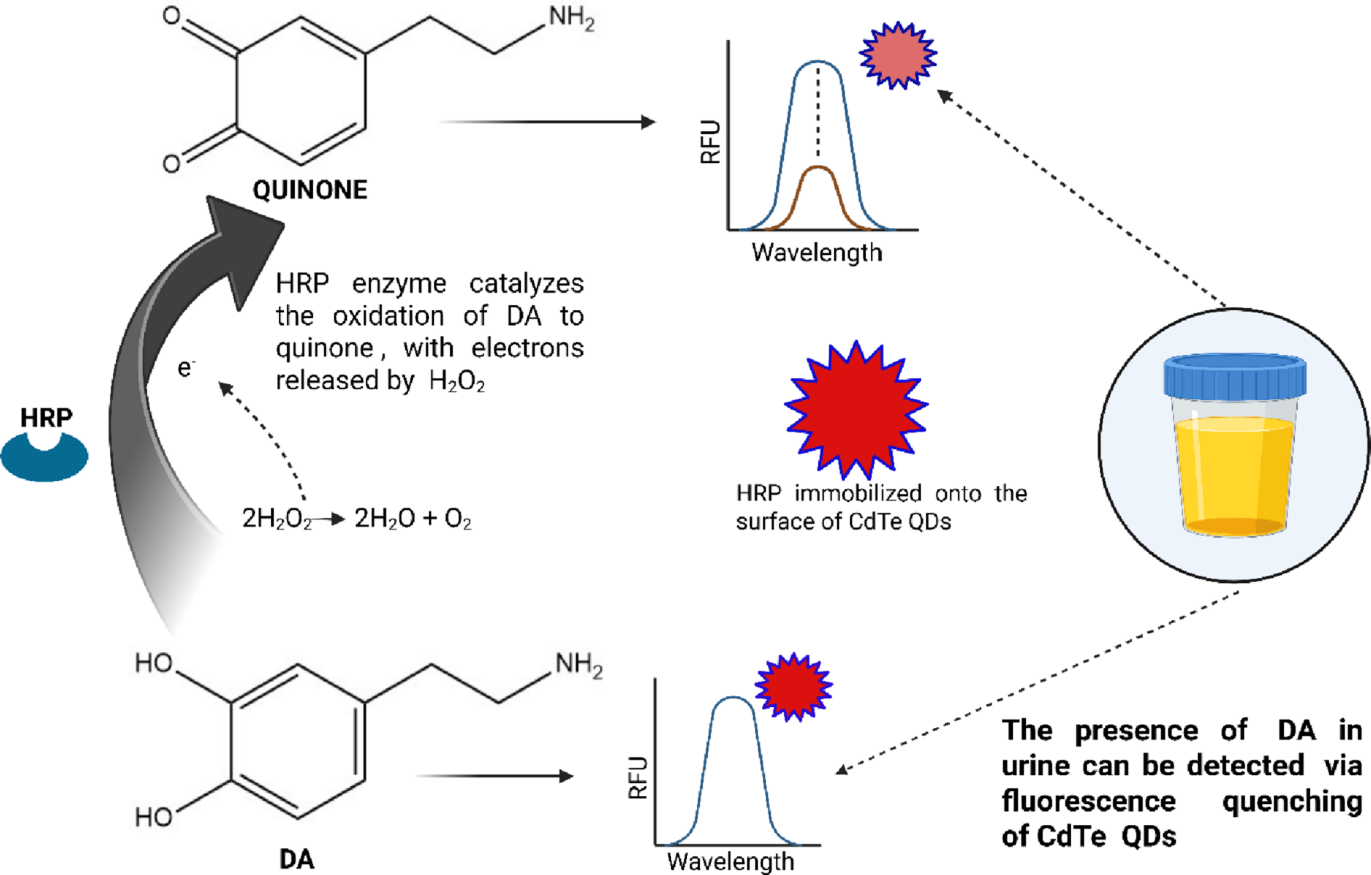
Scheme showing the conversion of DA to Quinone by HRP immobilized on CdTe QDs.

The low detection limit and reliability of fluorescent techniques^15^ have recently brought them to prominence. The fluorescent signals emitted by nanomaterials, such as surface-coated Quantum Dots (QDs) that are functionalized with biomolecules, such as enzymes, make them useful as biosensors^16^. Quantum dots, such as nitrogen-doped carbon dots (N-GQDs), are used for sensitive detection of DA in human urine and serum samples via fluorescence quenching, with an acceptable accuracy of > 80% an a detection limit of up to 4 nM^17^. A recent study using graphene quantum dots (GQDs) established a label-free fluorescence approach for sensitive DA detection. The addition of DA quenched the early blue fluorescence due to photoinduced electron transfer from boron sulfur co-doped QDs to DA-quinone. The fluorescence quenching of GQDs was linearly proportional to the increasing concentration of DA^18^. Also, GQD surfaces conjugated with polypyrrole enable sensitive detection of DA with a detection limit of 10 pM, where the interaction between polypyrrole’s amine group and DA’s hydroxyl group leads to a strong enhancement in fluorescence intensity^19^. An electrochemical biosensor, which is a novel imprinted polymer fabricated with NiS_2_ (Nickel disulfide) coated onto gold nanoparticles (AuNPs) and nitrogen-doped Graphene oxide quantum dots (N-GOQDs), is used for the sensitive and selective determination of DA in the nanomolar range with 93.9–106.15.9.15% recovery rate in hman urine, serum, and pharmaceutical samples^20^. In another study, Glutathione (GSH) protected gold nanoclusters (AuNCs) were used as fluorescent probes to detect DA. DA was oxidized to quinone in the presence of tyrosinase, which quenched the initial fluorescence of the AuNCs. In the presence of fluorescence intensity variation, a selective sensing assay was developed to detect DA with a LOD of 1 nM^21^. Polymer coating onto the surface of CdTe QDs exhibited considerable upconversion luminescence with two-photon excitation that aids in electron transfer from quinone to DA, which in turn was induced by DA anchored onto the surface of QD, leading to upconversion luminescence QD quenching. This method was able to detect DA down to a lower limit of 5.4 nM in biological fluids^22^. The work could not only provide insights into Tyrosinase and DA activity but also unlock potential strategies for tyrosinase inhibitor studies. However, the lack of evaluation using biological samples limits the practical applicability of these methods, as the matrix components could pose a challenge during detection. There are a few reports on electrochemical-based CdTe QD carbon electrodes for DA detection in urine^23^, QDs embedded in imprinted polymers^24^, and HRP-based systems for phenolic compounds^25^, with a focus mainly on H_2_O_2_-mediated quenching rather than DA as the primary analyte. To the best of our knowledge, the present work is the first fluorescence biosensing platform validated in the urine matrix. With this background, we have developed a Cadmium Telluride (CdTe) quantum dot-based enzyme biosensor to detect DA in the current study, as they have tunable emission across the visible spectrum such as broad absorption spectra and narrow emission spectra, highly sensitivity to different quenchers, enable water solubility, the presence of thiol group in mercaptopropionic acid in the cap facilitate surface functionalization of different ligands and offer good photostability that is resistant to photobleaching than other QDs^26^. In the presence of Horseradish peroxidase (HRP) and H_2_O_2_ as a substrate, DA oxidizes to its quinone form. This results in fluorescence quenching due to the interaction of the quinone form of DA and the CdTe QDs (Scheme 1). Electron acceptance of quinone by the excited state of QDs/electron transfer mechanisms like fluorescence resonance energy transfer (FRET)^27^ or adsorption of quinone molecules on the QD surface^28^ may alter their electron emission pattern and photoluminescent properties, thereby causing quenching. Using FRET, the quenching mechanism in which DA-quinone serves as the acceptor and CdTe QDs as energy donors is studied through non-radiative energy transfer. The interaction produces quinone species, which act as “dark quenchers” by accepting electrons or energy from QDs and decreasing their fluorescence. This non-radiative energy-transfer technique has been well established in our earlier report^29^. Based on this, the present study was conducted, in which the proposed method is a sensitive fluorescence-based method for detecting DA in a urine sample by quenching the fluorescence of CdTe QDs catalyzed by HRP^30^. In addition to establishing a reliable and sensitive platform for DA detection, the method is also validated by demonstrating practical applicability and efficacy in urine samples, showcasing its potential in diagnostics. DA detection using CdTe QDs that was doped on the surface of glassy electrode (GCE) - CdTe QDs/GCE showed an LOD of 300 nM, where there is no usage of enzyme and H_2_O_2_ in the presence of interferents such as uric acid (UA), glucose and ascorbic acid(AA)^31^, also the detection by molecular imprinted electrode (MIP) for DA detection exhibited selectivity against UA, AA and tyramine as interferents with an LOD of 650 nM^32^. While many existing sensing platforms exhibit high sensitivity, they are often susceptible to cross-reactivity because they lack biorecognition elements. The current method utilizes enzymes to ensure the necessary specificity for accurate detection in complex biological samples.

## Materials and methods

### Chemicals and reagents

All reagents were of analytical grade and used as received without further purification. Cadmium telluride Quantum Dots (CdTe QDs), core type QDs (λ_em_ 610 nm), Dopamine hydrochloride, and HRP were purchased from Sigma Aldrich, USA. Sodium phosphate dibasic heptahydrate and Sodium phosphate monobasic monohydrate for phosphate buffer (PB) preparation, and Hydrogen peroxide (H_2_O_2_) were purchased from Sigma Aldrich, USA. Urine samples for the study were collected from healthy volunteers after obtaining the necessary ethical clearance. Water used for reagent preparations was collected from the Millipore ultrapure system (Type 1). The data were collected using a Spectrofluorimeter (FP-8300, Jasco, Japan) for photoluminescence measurements.

### Enzyme assay

#### Optimization of enzyme concentration

To optimize HRP concentration, CdTe QDs (1 mg/mL) and DA (5 µM) were incubated for 5 min, followed by the addition of 300 µM H_2_O_2_ as a substrate. A varying concentration of HRP 2 U-12 U was prepared using phosphate buffer (1 mM PB, pH 7.4). In alkaline conditions, DA undergoes faster chemical autoxidation; however, pH 7.4 was chosen to maintain the structural integrity and optimize the efficiency of the HRP enzyme, which performs best at physiological levels. This setup ensures that the detected signal arises from enzymatic activity rather than non-specific base-catalyzed oxidation, thereby improving the assay’s selectivity and accuracy. The enzyme was added to the reaction mixture and incubated for 10 min at 37 °C. The Fluorescence spectra were recorded by exciting QDs (λ_ex_ 410 nm).

#### Optimization of H_2_O_2_ concentration

Optimized concentrations of QDs (CdTe QDs-1 mg/mL) were incubated with DA, followed by the addition of various concentrations of H_2_O_2_ (0.01 µM to 300 µM). All experiments were performed in triplicate (*n* = 3). In a separate experiment, CdTe QDs were incubated with various concentrations of H_2_O_2_ (10 nM-300 µM) to evaluate fluorescence quenching at a fixed concentration of the enzyme (6 U) and DA (5 µM), and fluorescence quenching was monitored for 10 min. H_2_O_2_ kinetics experiments were performed in triplicate (*n* = 3).

### Dopamine detection

DA at varying concentrations (1.2 µM- 4 µM) was prepared in PB, and the enzyme assay was performed using optimized enzyme and substrate concentrations. All experiments were performed in triplicate (*n* = 3). The reaction mixture was incubated for 10 min, and the fluorescence spectra were recorded.

#### Dopamine detection in urine samples

After obtaining ethical clearance from the central ethics committee (INST.EC/2023-24/001), DA detection was performed on urine samples. The interaction of urine samples with the proposed biosensing system was first evaluated. The urine samples were diluted (1:100 PB) to reduce the matrix effects. The urine samples were incubated with an optimized concentration of QDs, enzyme, and substrate, and fluorescence quenching was evaluated for 10 min (without DA). Further, DA at various concentrations (1.2 µM- 8 µM) was prepared and spiked into urine samples. As discussed earlier, the enzymatic assay was performed, and fluorescence spectra were recorded.

#### Spiking and recovery studies

Urine samples were collected from the donor and spiked with various DA concentrations (1.2 µM-8 µM), and the assay was performed as discussed in Sect. 2.3.1. The recovery percentage was calculated as provided in Table 1; all experiments were performed in triplicate (*n* = 3).

**Table 1:** DA detection in urine and recovery percentage.

Dopamine concentration (nM)	% Recovery
1200 nM	98.62%
2800 nM	96.81%
4000 nM	98.21%
7200 nM	94.25%

### Interference of uric acid, ascorbic acid, and different organic and inorganic constituents in urine in the detection of DA

The interference of uric acid and ascorbic acid at physiological normal concentrations was chosen to assess their impact on DA detection in urine samples. The urine samples were diluted (1:100 PB) to reduce the matrix effects. Uric acid kinetics were performed in triplicate with the concentrations ranging from 160 µM, 321 µM, and 441 µM, which were spiked to the urine sample with an optimized concentration of QDs, (6 U) enzyme, (300 µM) substrate, and fluorescence quenching, which was evaluated for 10 min (without DA), and fluorescence spectra were recorded. Similarly, ascorbic acid kinetics was performed at 283 µM, 425 µM, and 567 µM in triplicate. Interference of uric acid in the detection of DA was checked by spiking DA at 1.2 µM, 4 µM, and 8 µM to three different concentrations of uric acid, with respect to a control where no DA was spiked to uric acid. Followed by checking the interference of various organic (glucose) and inorganic constituents (sodium, sulfate, phosphate, potassium) present in the urine sample.

## Results and discussion

### Optimization of enzyme concentration

**Fig. 1:**
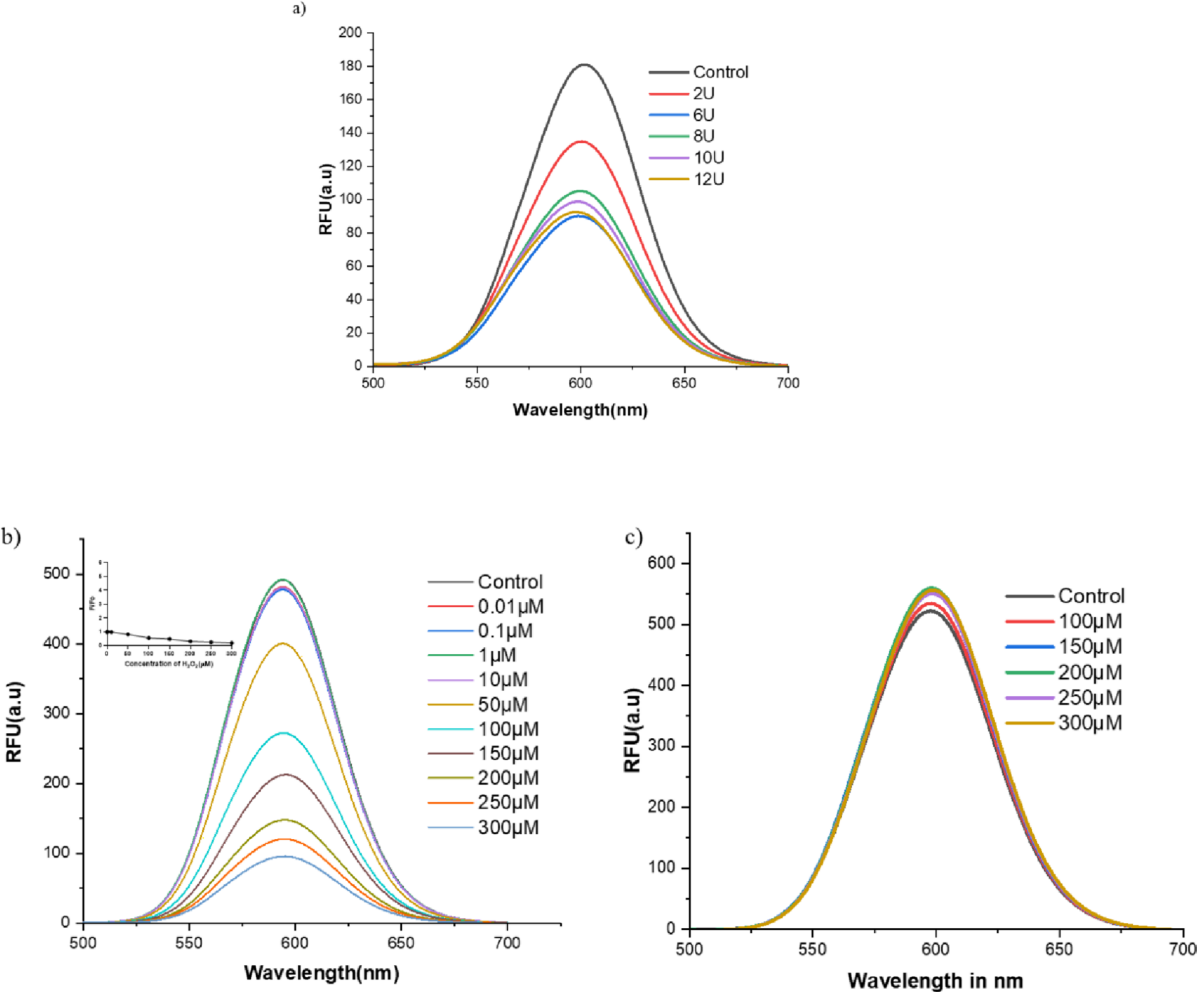
Enzyme Kinetics, H_2_O_2_ kinetics, and H_2_O_2_ interference spectra. (**a**) Fluorescence emission spectra of CdTe QDs in the presence of varying concentrations of HRP, from 6 U to 12 U, at a fixed DA (5 µM) and H_2_O_2_ concentration (300 µM). Control represents QD, H_2_O_2_, and HRP with no DA in PB. Increasing the concentration of HRP results in enhanced fluorescence quenching until the enzyme reaches its saturation. (**b**) Fluorescence emission spectra of CdTe QDs in the presence of varying concentrations of H_2_O_2_ substrate, from 0.01 µM to 300 µM, at fixed DA (5 µM) and HRP (6 U). Control represents QD, H_2_O_2_, and HRP with no DA in PB. Increasing the concentration of H_2_O_2_ results in enhanced fluorescence quenching until it reaches saturation. (**c**) H_2_O_2_ interference with the fluorescence intensity of CdTe QDs in the presence of varying concentrations of H_2_O_2_ substrate only, from 0.01 µM to 300 µM, in the absence of enzyme and substrate with respect to the control.

HRP, an oxidoreductase, catalyzes the oxidation of DA in the presence of H_2_O_2_^33^. To optimize the enzyme concentration required for the oxidation of DA to dopaquinone in the presence of H_2_O_2_, an enzyme kinetic assay was performed. As depicted in Fig. 1a, an increasing concentration of HRP from 6 U to 12 U resulted in a progressive reduction in relative fluorescence intensity. The quenching fluorescence ratio (F/Fo) of CdTe QDs was in proportion to the HRP concentrations (F/Fo 1.35–0.90, HRP 6 U-12 U). A maximum fold decrease in fluorescence (F/Fo = 0.88) was observed at 6 U, and further changes were not significant. Based on these results, since maximum fluorescence quenching is observed at 6 U HRP concentration, which is fixed for the rest of the study, suggesting the enzyme has reached its maximal catalytic turnover rate under fixed substrate concentration (300 µM), consistent with Michaelis-Menten saturation behavior^34^. The spectral trend indicates that 6U HRP was sufficient for near-complete oxidation of 5 µM DA under the set experimental conditions. A kinetic profile similar to this was observed in a study in which phenol was oxidized by HRP, following an irreversible Michaelis-Menten reaction in the presence of H_2_O_2_, thereby identifying the rate-limiting step^35^. This aligns with the observation that reaction rates increase with substrate concentration but level off once the enzyme is saturated. There will be a substantial shift in the reaction rate, as observed in the kinetics curve, after the rate reaches its saturation point^36^, leading to the oxidation of DA to quinone. DA oxidation to quinone is observed by fluorescence quenching of CdTe QDs, which increases with increasing incubation time^37^. CdTe QDs were used at a working concentration corresponding to the 1 mg/mL stock per reaction, which was empirically found to provide sufficiently high fluorescence intensity without detector saturation, and to allow clear observation of DA‑induced quenching.

### Optimization of H_2_O_2_ concentration and H_2_O_2_ interference

**Fig. 2:**
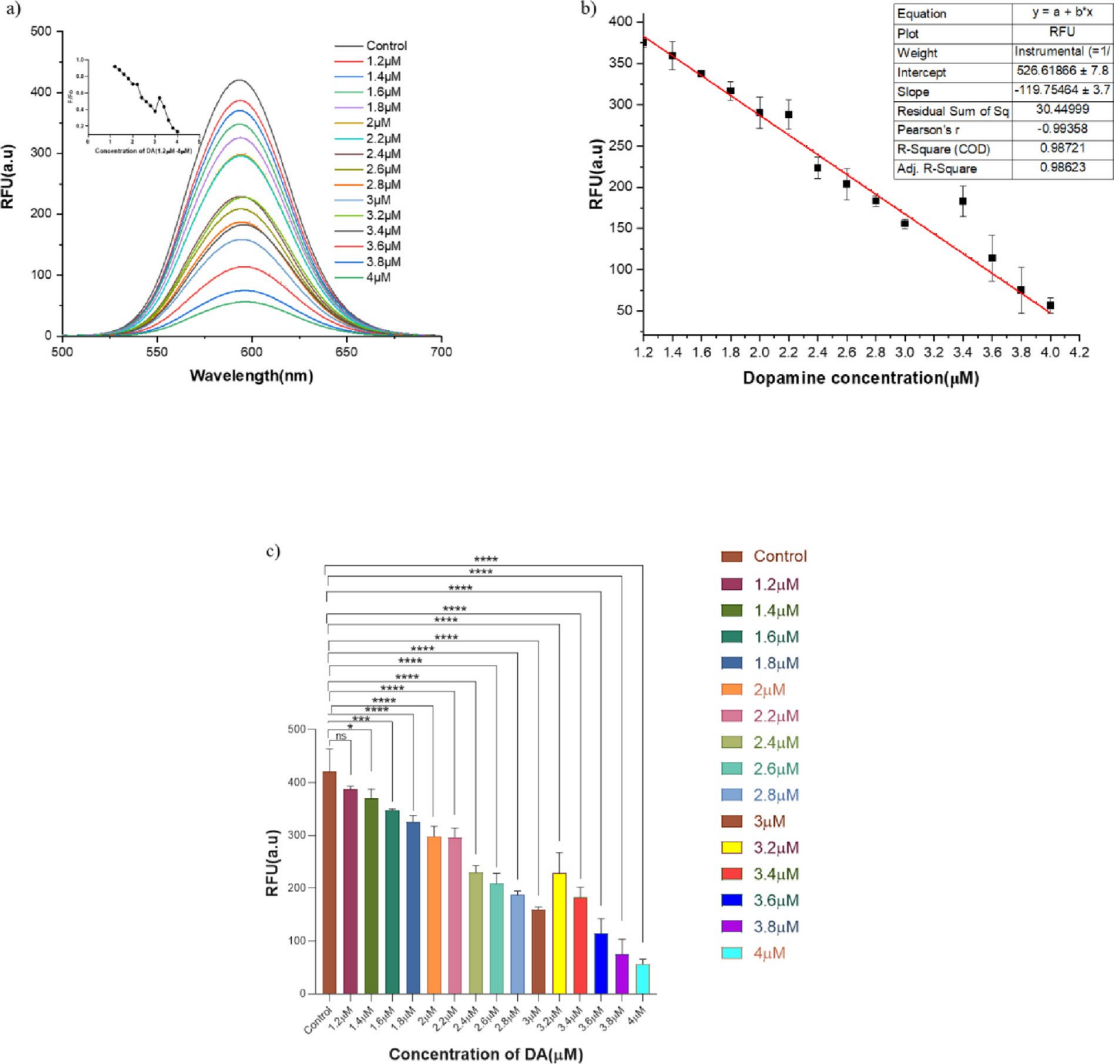
DA kinetics in an aqueous system. (**a**) Fluorescence emission spectra of CdTe QDs in the presence of varying concentrations of DA, from 1.2 µM to 4 µM, at a fixed H_2_O_2_ (300 µM) and HRP (6 U). Control represents QD, H_2_O_2_, and HRP with no DA in PB. Increasing the concentration of dopamine results in enhanced fluorescence quenching until it reaches saturation. (**b**) DA standard deviation graph showing concentration-dependent fluorescence quenching from 1.2 µM to 4 µM with a fixed H_2_O_2_ (300 µM) and HRP (6 U), having an R^2^ = 0.99. (**c**) Bar graph indicating fluorescence intensity (RFU) measured in an aqueous system with increasing concentrations of dopamine (1.2 µM to 4 µM). A gradual decrease in fluorescence intensity is observed with increasing dopamine concentration, indicating effective quenching by DA. Data were represented as mean ± SD (*n* = 3). Statistical analysis was performed using one-way ANOVA, followed by Dunnett’s multiple-comparison test against the control group, with statistical significance (*p* < 0.05) relative to the control.

H₂O₂ is a common oxidant produced by oxidases. Its unstable bonds lead to breakdown, forming hydroxyl ions or radicals used in sensing. Reaction conditions influence the formation of hydrogen and oxygen. Active sites in the sensor catalyze the formation of OH• radicals, driving H₂O₂ degradation^38,39^. H_2_O_2_ breakdown releases electrons that are used in the conversion of DA to Quinone, serving as a substrate utilized in the present study, analyzed by fluorescence quenching of CdTe QDs. O₂(aq)/H₂O₂ and DQ/DdxQH₂ (catechol/quinone moiety) redox potentials show that the two-electron oxidation of DA by the oxygen/hydrogen peroxide redox pair is thermodynamically advantageous at all pH values^40^. Fluorescence quenching of CdTe QDs for the breakdown of the substrate H_2_O_2_ is observed in the ratio (F/Fo), which was in alliance with the lowest (0.01 µM) to the highest concentration of H_2_O_2_ (300 µM). The fluorescence quenching ratio (F/Fo = 0.19) for the highest concentration of H_2_O_2_ (300 µM) and (F/Fo = 0.98) was for the lowest (0.01 µM) H_2_O_2_. It is important to note that the highest concentration of H_2_O_2_ (300 µM) significantly reduces the intensity, showing a threshold effect (Fig. 1b). Figure 1b shows a control with QD, H_2_O_2_, and HRP without DA; Fig. 1c shows a control with QD in PB. At 0.01 µM concentration of H_2_O_2_, fluorescence quenching ratio (F/Fo) for 0.01 µM H_2_O_2_ concentration was 0.98 in comparison to the highest concentration (300 µM) (F/Fo = 0.195), suggesting that a low concentration of H_2_O_2_ maintains intensity, having a very minimal effect on the intensity of the reaction. The highest H_2_O_2_ concentration shows a greater effect in the presence of 6 U of HRP enzyme concentration, CdTe QDs, and 5 µM DA concentration, all dissolved in PB of pH 7.4. Among the concentrations ranging from 0.01 µM-300 µM of H_2_O_2_ tried in the presence of HRP enzyme and DA, there is only a minimal effect observed at 0.01 µM H_2_O_2_ concentration and a maximum effect observed at 300 µM H_2_O_2_. Based on these results, 300 µM H_2_O_2_ was determined to be the optimal concentration for the reaction. In the absence of HRP and DA in the reaction mixture, there is little effect on the fluorescence quenching of H_2_O_2_ kinetics, as shown in Fig. 2b. The kinetics was carried out only in PB and QD with varying concentrations of H_2_O_2_, fluorescence quenching ratio (F/Fo = 1.055) for 300 µM H_2_O_2_. This states that, for fluorescence quenching to occur, the interaction of H_2_O_2_ with HRP (6 U) and DA (5 µM) is necessary^41,42^.

### Dopamine biosensing

After fixing the H_2_O_2_ concentration and reaction time, different DA concentrations (1.2 µM to 4 µM) dissolved in MilliQ water were tested. Oxidation of DA to quinone in the presence of H_2_O_2_ as a substrate occurs on the surface of the HRP enzyme, which is analyzed by fluorescence quenching of QDs^43,44^. The reaction mixture consists of 6 units of HRP, 300 µM H_2_O_2,_ and different concentrations of DA in PB at pH 7.4, where concentration-dependent fluorescence quenching was observed, excited at λ_ex_ 420 nm. The highest concentration of DA (4 µM) showed a fluorescence quenching ratio (F/Fo = 0.137) with respect to the control intensity, and a (F/Fo = 0.92) fluorescence quenching ratio for 1.2 µM of DA compared with the control was observed after 10 min of incubating all the reaction components (Fig. 2a). Figure 2 consists of controls with QD, H_2_O_2_, and HRP with no DA. Oxidation of DA to quinone is due to the presence of H_2_O_2_, which releases electrons that are utilized in the oxidation step in the presence of HRP as an enzyme^30^. This HRP-based QD biosensor had a detection limit of 1.2 µM and showed activity with a linear range of 1.2 µM to 4 µM^45^. The relationship between fluorescence intensity and DA concentration was plotted as a standard deviation graph with (R^2^ = 0.99) (Fig. 2b), indicating that the developed method is sensitive and reliable. Also, one-way ANOVA was performed, which showed a *p* < 0.0001 from 1.8 to 4 µM, showing statistical significance (Fig. 2c).

### Different dilutions of urine in dopamine biosensing

**Fig. 3:**
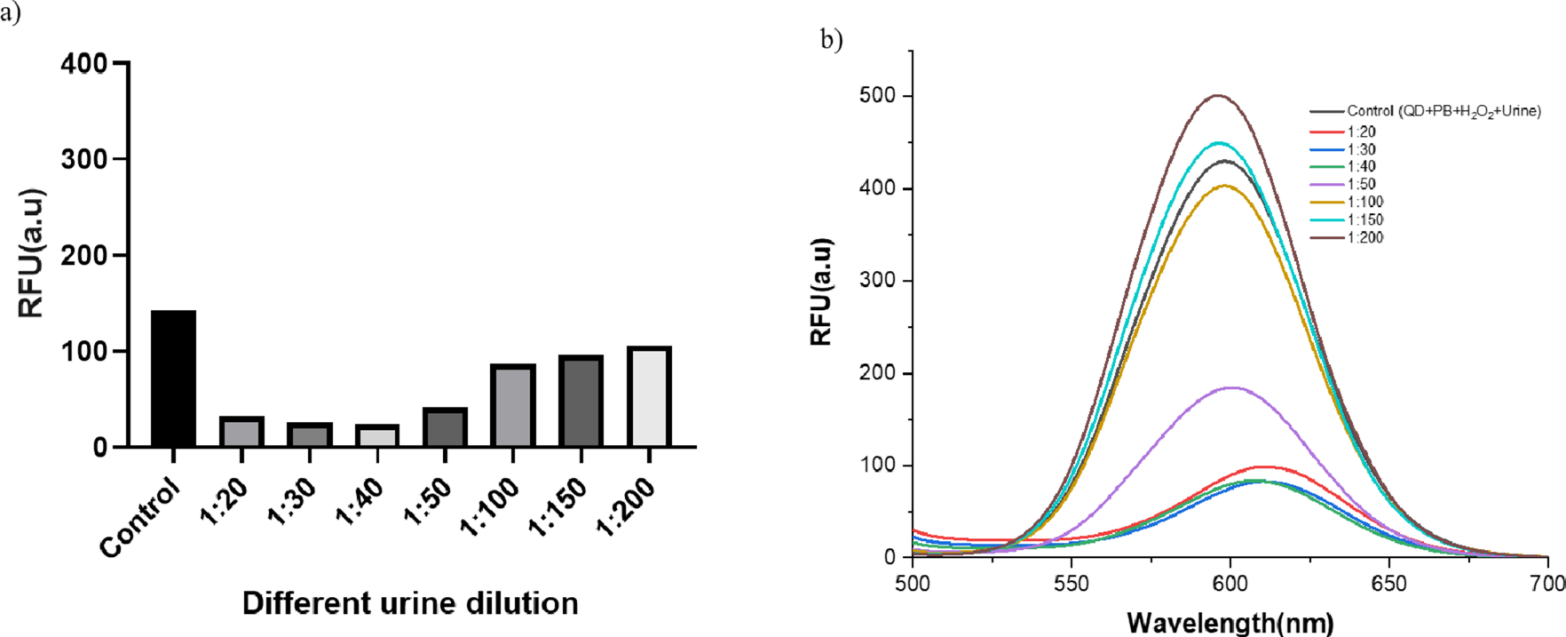
Fluorescence of CdTe QD-based dopamine biosensor in different dilutions of urine sample, ranging from 1:20,1:30,1:40,1:50,1:100,1:150, and 1:200 against a control having QD and PB (a), which was compared against a control having QD, PB, H_2_O_2_, and (1:100) urine. HRP.

In order to investigate the interference of urine components in DA biosensing through matrix effect, urine sample from a healthy individual was diluted in PB buffer at various ratios where 1 part is urine sample diluted in 19 parts of PB v/v, including 1:20, 1:30, 1:40, 1:50, 1:100, 1:150, and 1:200. The interference of components of urine was checked in the reaction mixture with control’s having the enzyme (HRP) and substrate (H_2_O_2_) without DA. Figure 3a depicts the spectra of different urine dilutions having QD in PB as a control, and Fig. 3b depicts the spectra of different urine dilutions having only H_2_O_2_ along with QD in PB as a control. At 1:20 − 1:50, we observed a clear reduction in fluorescence upon treatment with the reaction mixture, which could be attributed to the urine matrix. To reduce non-specific signals, we used a 1:100 dilution factor and did not further dilute the urine sample.

### Dopamine biosensing in urine samples

**Fig. 4:**
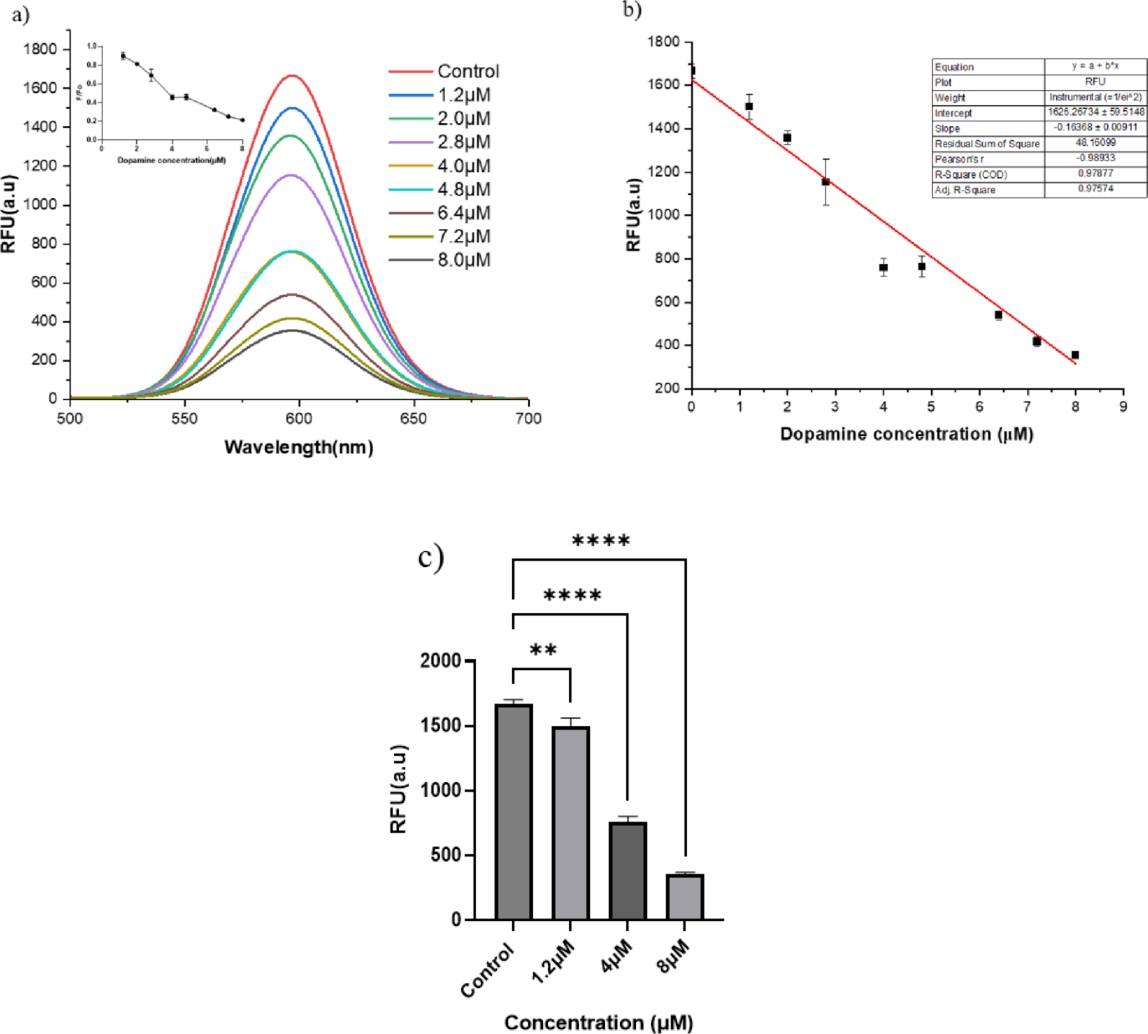
DA biosensing in urine. (**a**) Fluorescence emission spectra of CdTe quantum dots-based DA biosensor in spiked urine samples, with DA concentration varying from 1.2 to 8 µM at a fixed H_2_O_2_ (300 µM) and HRP concentration (6 U). A progressive quenching of fluorescence with increasing DA concentration is observed due to the oxidation of DA and subsequent interaction with the CdTe QDs. The DA standard deviation graph shows concentration-dependent fluorescence quenching from 1.2 µM to 8 µM with fixed H_2_O_2_ (300 µM) and HRP (6 U), with R^2^ = 0.97. (**b**) The DA standard deviation graph shows concentration-dependent fluorescence quenching from 1.2 µM to 8 µM with a fixed H_2_O_2_ (300 µM) and HRP (6 U), with an R^2^ = 0.97. (**c**) Bar graph indicating the detection of DA in spiked urine samples. The bar graph shows fluorescence intensity (RFU) measured after treating urine samples spiked with increasing concentrations of DA (1.2 µM to 8 µM). A gradual decrease in fluorescence intensity with increasing DA concentration indicates effective quenching. Data are represented as mean ± SD (*n* = 3). Statistical analysis was performed using one-way ANOVA, followed by Dunnett’s multiple-comparison test against the control group, with statistical significance (*p* < 0.05) relative to the control.

**Fig. 5:**
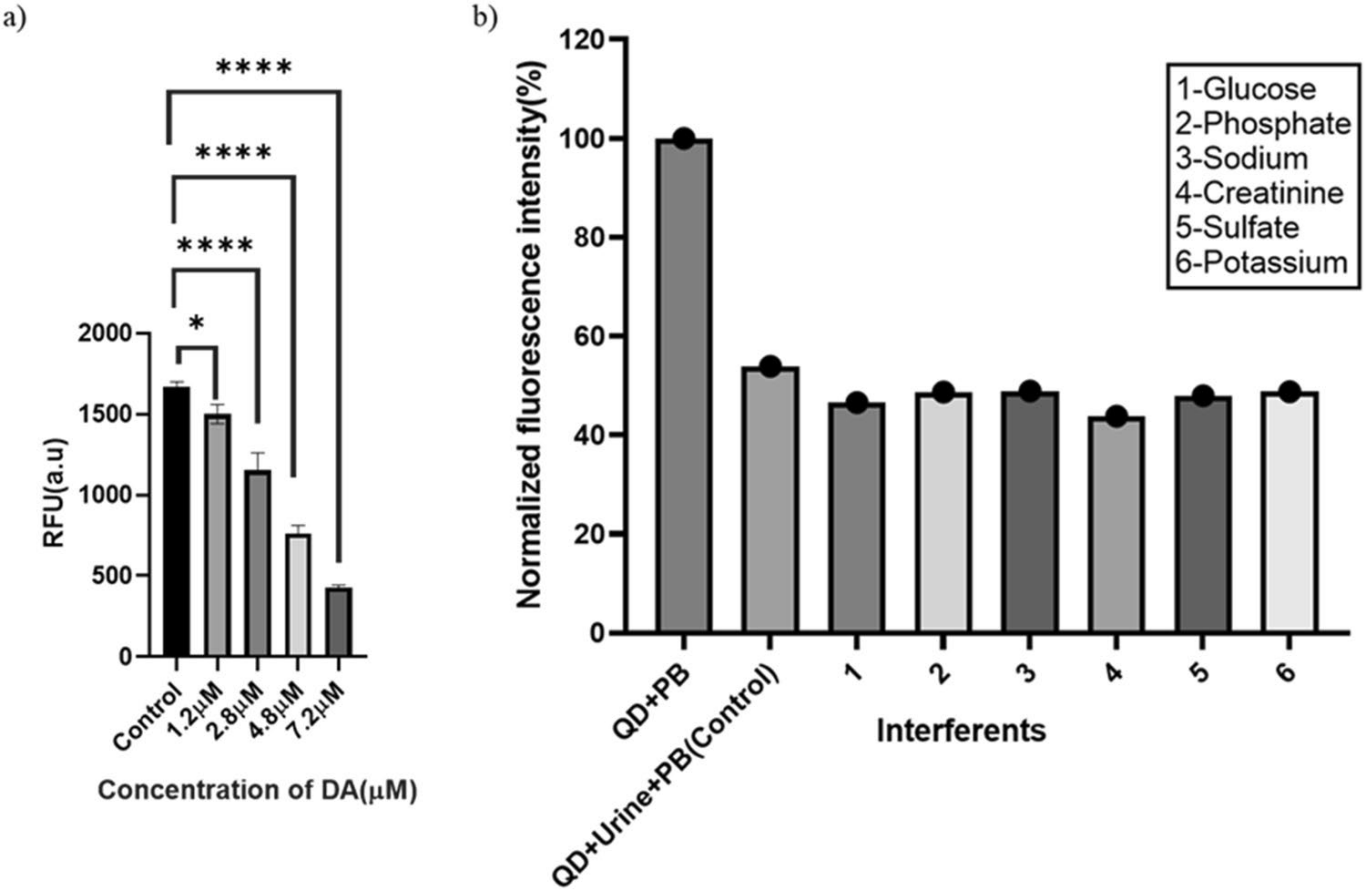
**(a**) Spike and recovery of DA and (**b**) Interference of organic and inorganic constituents in the Urine sample. (**a**) Bar graph indicating detection and recovery with different DA concentrations (1.2 µM-7.2 µM). Data represent mean ± SD of three independent replicates (*n* = 3). Statistical analysis was performed using one-way ANOVA, followed by Dunnett’s multiple comparisons test against the control group, with statistical significance (*p* < 0.05) compared to the control. (**b**) The interference of organic constituents such as Glucose (416.29 µM) and creatinine (70.72 µM), and inorganic constituents such as phosphate (0.03 µM), sodium (0.1 µM), sulphate (0.03 µM), and potassium (0.125 µM) in the urine sample was checked at a fixed H_2_O_2_ (300 µM) and HRP concentration (6 U).

The fluorescence quenching of DA was evaluated in DA-spiked urine samples using a quantum dot–enzyme-based biosensor system. Figure 4a shows a systematic decrease in relative fluorescence intensity with increasing DA concentration from 1.2 µM to 8 µM under reaction conditions similar to those in the aqueous system. Fold decrease(F/Fo) was used to quantify the degree of quenching. Compared with the control, F/Fo values declined progressively from 0.90 at 1.2 µM to 0.18 at 8 µM. The kinetic behavior is consistent with previous results on the detection of DA in aqueous systems, validating that the biosensing platform retains its functionality even in complex matrices. A recovery study was conducted using urine samples spiked with varying DA concentrations (1.2 µM-7.2 µM), showing % recovery ranging from 94.25% to 98.62%, emphasizing the analytical accuracy and minimal matrix-induced suppression effects (Table 1). The level of recovery is consistent with prior reports with fluorescent nanoparticle-based biosensors for DA detection in biological fluids^46–48^. The linear standard curve (R^2^ = 0.97877) with a strong negative correlation (Pearson’s *r* = −0.98933) between relative fluorescence intensity and DA concentration (Fig. 4b) indicates the reliability and sensitivity of the developed biosensor, which can be further extended to multiplexed detection in other body fluids. Detection of DA in spiked urine samples was compared with that of control at 4 µM and 8 µM (Fig. 4c). While Fig. 5a explains the spike and recovery of DA in the urine sample, which is analyzed using one-way ANOVA. As DA exists as a free base in biological fluids such as urine, 65–400 µg/24 h (0.42–2.61 µM for 1 L urine), which was studied in a healthy population, where 521.7 µg/24 h (3.4 µM) is the highest DA concentration due to dietary intake and the subject being studied^49^. To ensure clinical relevance, the sensor’s analytical performance was validated in a real human urine matrix, accounting for potential matrix effects absent in aqueous standards. The testing range was extended to 8.0 µM to encompass the full clinical spectrum, bridging the gap between the healthy physiological maximum ~ 3.4 µM^49^ and pathological hyper-excretion levels observed in conditions such as metastatic paraganglioma, which can reach 3187.5 µg/day (~ 20.8 µM)^50^. The linear quantification range is optimized to 4.0 µM in aqueous samples; however, the sensor remains highly effective for detecting pathological hyperexcretion of DA up to 8.0 µM.

### Interference of uric acid, ascorbic acid, and different organic and inorganic constituents in urine in the detection of DA

**Fig. 6:**
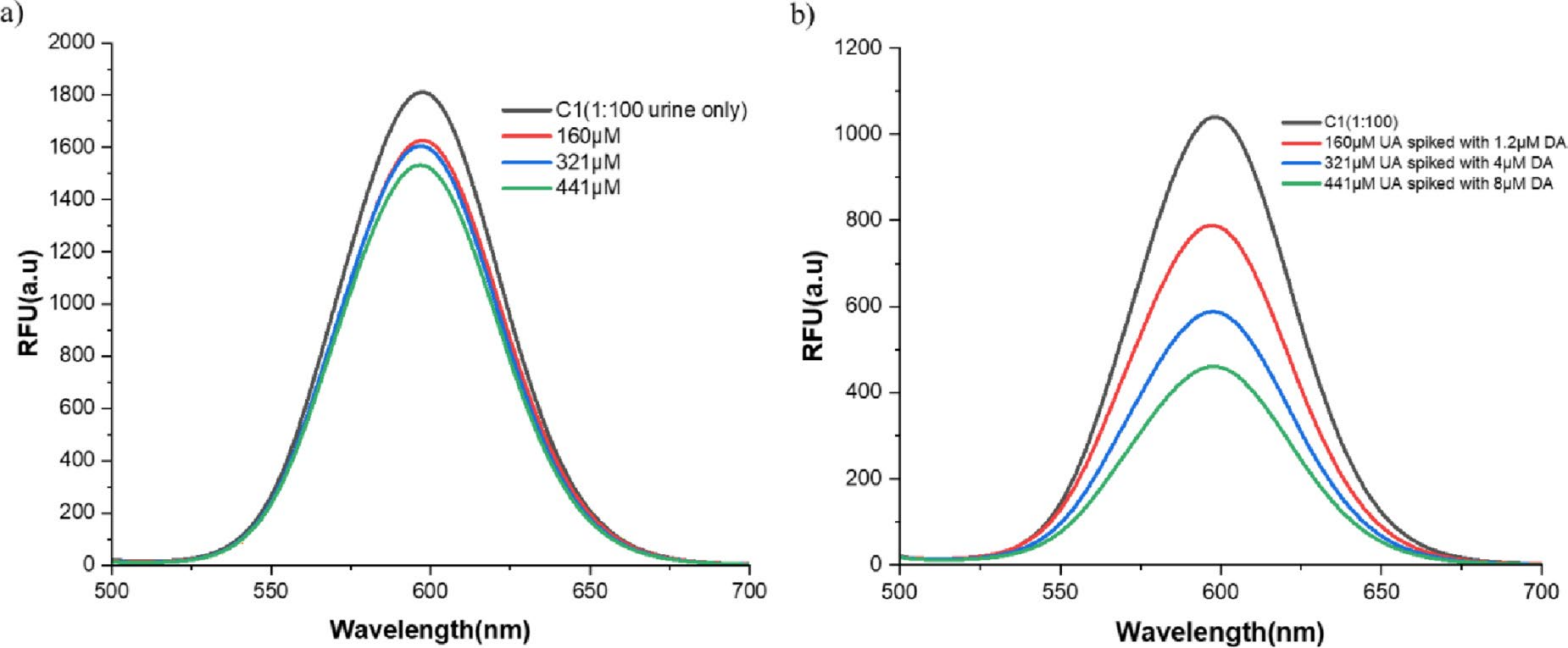
**(a**) UA kinetics in urine and (**b**) DA interference with Uric acid in a urine sample. Fluorescence emission spectra of CdTe quantum dots-based DA biosensor for different UA concentrations spiked to urine samples, with UA concentration varying from 160 µM, 321 µM, and 441 µM (**a**) and for different DA concentrations spiked to UA in urine samples, with DA concentration varying from 1.2 µM, 4 µM, and 8 µM, were spiked to UA of concentrations varying from 160 µM, 321 µM, and 441 µM at a fixed H_2_O_2_ (300 µM) and HRP concentration (6 U).

The standard graph and recovery studies for DA detection were performed on the urine sample to avoid interference from commonly occurring urine components. As depicted in Fig. 5b, ~ 50% fluorescence quenching was observed with the QD+urine+enzyme system, which can be attributed to HRP-mediated oxidation of interfering molecules. The control was normalized, and varying DA concentrations were added, and the biosensor response was evaluated. Nevertheless, to show biosensor robustness, we spiked various organic and inorganic constituents into the urine sample (1:100), and the fluorescence quenching pattern was evaluated. We observed that uric acid (UA of 160–441 µM) can quench fluorescence (Fig. 6a)^51^. It is important to note that uric acid alone showed 10–15%quenching of the fluorescence, nevertheless, upon addition of 1.2 µM/8 µM of DA the quenching was further increased in response to enzyme reaction to ~ 25–56%showing the robustness of biosensor (Fig. 6b). Additionally, the broad-spectrum interference study involving major electrolytes (Na^+^, K^+^, SO_4_^2−^, PO_4_^3−^) and metabolites (glucose, creatinine) showed a negligible interference (Fig. 5b).

## Conclusion

The CdTe QD-based fluorescence biosensor provides an effective and sensitive platform for detecting DA. Systematic standardization of substrate, enzyme, and reaction time revealed that 300 µM H_2_O_2_, 6 U HRP, and 10 min of incubation are optimal for maximal quenching of fluorescence and subsequent DA detection via oxidation of DA to dopaquinone. The real-world applicability of the biosensor was validated using DA-spiked urine samples, which showed a remarkable detection response from 1.2 µM to 8 µM of DA, with recovery percentages ranging from 94.25% to 98.62%. A strong correlation between different DA levels and relative fluorescence intensity underscores the nanosensor’s reliability and potential, paving the way for multiplexed biosensing in other biological fluids.

## Data Availability

All data generated or analysed during this study are included in this article.

## Abbreviations

DA: Dopamine
TH: Tyrosine hydroxylase
AuNPs: Gold nanoparticles
GSH: Glutathione
AuNCs: Gold nanoclusters
QDs: Quantum Dots
HPLC: High performance liquid chromatography
ELISA: Enzyme-linked immune sorbent assay
CdTe: Cadmium Telluride
FRET: Fluorescence resonance energy transfer
N-GOQDs: Nitrogen doped -Graphene oxide quantum dots
HRP: Horseradish peroxidase
UA: Uric acid
AA: Ascorbic acid
GCE: Surface of glassy electrode
